# *In Vitro* and *In Vivo* Characterization of H5N8 High-Pathogenicity Avian Influenza Virus Neurotropism in Ducks and Chickens

**DOI:** 10.1128/spectrum.04229-22

**Published:** 2023-01-10

**Authors:** Charlotte Foret-Lucas, Thomas Figueroa, Amelia Coggon, Alexandre Houffschmitt, Gabriel Dupré, Maxime Fusade-Boyer, Jean-Luc Guérin, Maxence Delverdier, Pierre Bessière, Romain Volmer

**Affiliations:** a Ecole Nationale Vétérinaire de Toulouse, Université de Toulouse, ENVT, INRAE, IHAP, UMR 1225, Toulouse, France; Utrecht Institute for Pharmaceutical Sciences, Utrecht University

**Keywords:** chicken, duck, highly pathogenic avian influenza, influenza, neuron, neurotropism, primary

## Abstract

H5N8 high-pathogenicity avian influenza virus (HPAIV) of clade 2.3.4.4B, which circulated during the 2016 epizootics in Europe, was notable for causing different clinical signs in ducks and chickens. The clinical signs preceding death were predominantly neurological in ducks versus respiratory in chickens. To investigate the determinants for the predominant neurological signs observed in ducks, we infected duck and chicken primary cortical neurons. Viral replication was identical in neuronal cultures from both species. In addition, we did not detect any major difference in the immune and inflammatory responses. These results suggest that the predominant neurological involvement of H5N8 HPAIV infection in ducks could not be recapitulated in primary neuronal cultures. *In vivo*, H5N8 HPAIV replication in ducks peaked soon after infection and led to an early colonization of the central nervous system. In contrast, viral replication was delayed in chickens but ultimately burst in the lungs of chickens, and the chickens died of respiratory distress before brain damage became significant. Consequently, the immune and inflammatory responses in the brain were significantly higher in duck brains than those in chickens. Our study thus suggests that early colonization of the central nervous system associated with prolonged survival after the onset of virus replication is the likely primary cause of the sustained inflammatory response and subsequent neurological disorders observed in H5N8 HPAIV-infected ducks.

**IMPORTANCE** The severity of high-pathogenicity avian influenza virus (HPAIV) infection has been linked to its ability to replicate systemically and cause lesions in a variety of tissues. However, the symptomatology depends on the host species. The H5N8 virus of clade 2.3.4.4B had a pronounced neurotropism in ducks, leading to severe neurological disorders. In contrast, neurological signs were rarely observed in chickens, which suffered mostly from respiratory distress. Here, we investigated the determinants of H5N8 HPAIV neurotropism. We provide evidence that the difference in clinical signs was not due to a difference in neurotropism. Our results rather indicate that chickens died of respiratory distress due to intense viral replication in the lungs before viral replication in the brain could produce significant lesions. In contrast, ducks better controlled virus replication in the lungs, thus allowing the virus to replicate for a sufficient duration in the brain, to reach high levels, and to cause significant lesions.

## INTRODUCTION

High-pathogenicity avian influenza virus (HPAIV) outbreaks are a major concern both for animal and public health. Over the past years, clade 2.3.4 H5Nx HPAIVs have gradually become predominant across the world. Clade 2.3.4.4B H5N8 virus has efficiently spread through migratory bird pathways and recently caused the first H5N8 human infections (1–5). This virus has caused unprecedented epizootics in Europe, resulting in huge economic losses and in a tremendous number of poultry culled.

In order to become fusogenic, the viral protein hemagglutinin (HA) has to be cleaved into two subunits, namely, HA1 and HA2 (6). The HA of low-pathogenicity influenza viruses (LPAIVs) is proteolytically activated by trypsin-like proteases expressed in the respiratory and the digestive tracts. In contrast, due to its polybasic cleavage site, the HA of HPAIV can be cleaved by ubiquitous proteases, thus allowing systemic viral spread. In chickens, HPAIV infections result in high morbidity and mortality. On the contrary, HPAIV infections are generally mild or asymptomatic in ducks (7–10). One of the distinguishing features of clade 2.3.4.4B HPAIV H5N8 is that it can cause severe disease in wild birds and waterfowl, including ducks (10–12).

Depending on viral and host factors, disease caused by HPAIV infection may vary from sudden death to various clinical presentations, including neurological signs. Notably, field observations and experimental infections indicate that clade 2.3.4.4B H5N8 virus has a pronounced neurotropism in ducks, leading to severe neurological disorders, such as ataxia or convulsion (11, 13–16). In contrast, neurological signs are rarely observed in *Galliformes*, which suffer mostly from respiratory distress (11, 13–16). These elements prompted us to investigate the ability of clade 2.3.4.4B H5N8 HPAIV to infect and replicate in the central nervous system, by comparing chickens and ducks using *in vitro* and *in vivo* models.

## RESULTS

### H5N8 replication in primary neurons.

**FIG 1:**
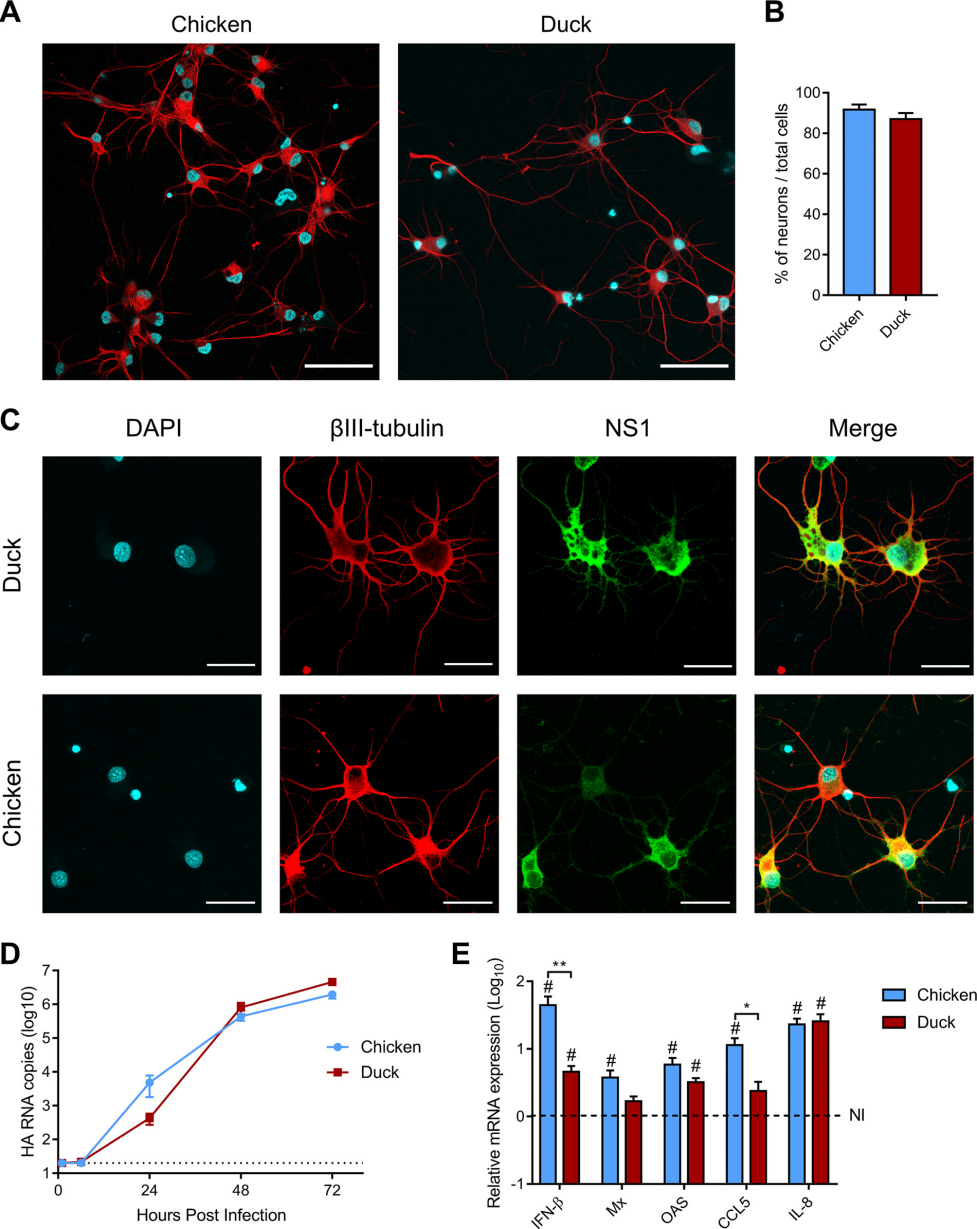
H5N8 HPAIV infections in primary neurons. (A and B) Assessment of chicken and duck cortical neuron morphology and purity. (A) Immunofluorescence staining of neurons after 4 days of culture with βIII-tubulin (red) and DAPI (blue). Scale bar, 50 μm. (B) Percentage of neurons, calculated as the ratio of the number of βIII-tubulin-stained neurons to the total number of DAPI-stained nuclei, from 10 randomly selected microscope fields for each species. (C) Immunofluorescence staining of chicken and duck neurons infected at an MOI of 2 and fixed 8 h postinfection. NS1, green; βIII-tubulin, red; DAPI, blue. Scale bar, 25 μm. (D) Viral replication kinetics. Neurons from both species were infected at an MOI of 10^−4^ TCID_50_. HA RNA load was analyzed by RT-qPCR. Data are displayed as mean ± standard error of the mean (SEM) from three independent experiments. Statistical analysis was performed used two-tailed Mann-Whitney test. The dotted line represents the limit of detection. (E) Immune and inflammatory marker mRNA expression following chicken and duck neuron infection. Neurons from both species were infected at an MOI of 2. mRNA expression levels of IFN-β, Mx, OAS, CCL5, and IL-8 were determined by RT-qPCR performed on chicken and duck neuron total RNA. mRNA levels were normalized using the 2^ΔΔ−^*^CT^* method. Data are displayed as mean ± standard error of the mean (SEM) from three independent experiments. Statistical analysis used the two-tailed Mann-Whitney test. The dotted line represents mRNA expression levels of noninfected (NI) neurons. Results are expressed as means ±SEM. *, *P* < 0.05; **, *P* < 0.01; #, *P* < 0.05 compared to noninfected neurons.

The preponderance of neurological symptoms in H5N8 HPAIV-infected ducks could be due to an increased permissiveness of duck neurons to H5N8 HPAIV infection. We therefore compared the permissiveness of duck and chicken primary cortical neurons to that of H5N8 HPAIV. Neurons isolated from cerebral cortexes represented approximatively 90% of all cells in duck and chicken cultures, as assessed by βIII-tubulin staining, and displayed typical neuronal morphology (Fig. 1A and B). To verify that cortical neurons of both species were permissive to H5N8 infection, they were infected with H5N8 at a multiplicity of infection (MOI) of 2. Cells were stained with an anti-influenza protein NS1 antibody at 8 h postinfection. Staining results confirmed that the virus could infect both chicken and duck neurons (Fig. 1C). We then analyzed the levels of virus replication in neurons infected at a low MOI (10^−4^). No difference was found between the two species (Fig. 1D), showing that H5N8 HPAIV replicated similarly in chicken and duck neurons. To determine if the polybasic HA cleavage site was essential for neuronal replication, we used an H5N8 LPAIV differing solely at the level of the HA cleavage site, as described previously (14). In the presence of exogenous trypsin, H5N8 LPAIV replicated to similar levels as H5N8 HPAIV, so long as the growth medium did not contain the B-27-plus supplement, which inhibited extracellular protease activity (data not shown).

To explore the consequences of the infection on the immune response, we analyzed the expression of innate immunity and inflammation genes in neurons infected with H5N8 HPAIV at an MOI of 2 (Fig. 1E). We quantified viral replication levels by measuring intracellular HA viral RNA. Viral RNA levels were equivalent in chicken and duck neurons at 24 h postinfection, indicating similar levels of replication (data not shown). mRNA levels of interferon beta (IFN-β), Mx, OAS, CCL5, and interleukin-8 (IL-8) were significantly increased in H5N8 HPAIV-infected chicken neurons compared with those in noninfected ones. In contrast, only IFN-β, OAS, and IL-8 mRNA levels were significantly increased in H5N8 HPAIV-infected duck neurons. We detected an increase in IFN-β and CCL5 mRNA expression in infected chicken neurons compared with those in infected duck neurons (*P* < 0.01 and *P* < 0.05, respectively). Although the immune response to H5N8 HPAIV was lower in duck neurons, the virus replicated to similar levels in duck and chicken neurons, suggesting that the factors contributing to the increased neurotropism in ducks compared with those in chickens may not be identifiable *in vitro*.

### H5N8 replication in chickens and ducks.

To explore the determinants of H5N8 HPAIV neurotropism *in vivo*, we analyzed archived samples from two *in vivo* experiments carried out in ducks and chickens, respectively, which have been described previously (14), and from two unpublished experiments, carried out in ducks and chickens, respectively. We thus present pooled data from two independent experiments in ducks and two independent experiments in chickens, which were all carried out in 4-week-old birds infected with 10^4^ 50% egg infective dose (EID50) of H5N8 HPAIV.

**FIG 2:**
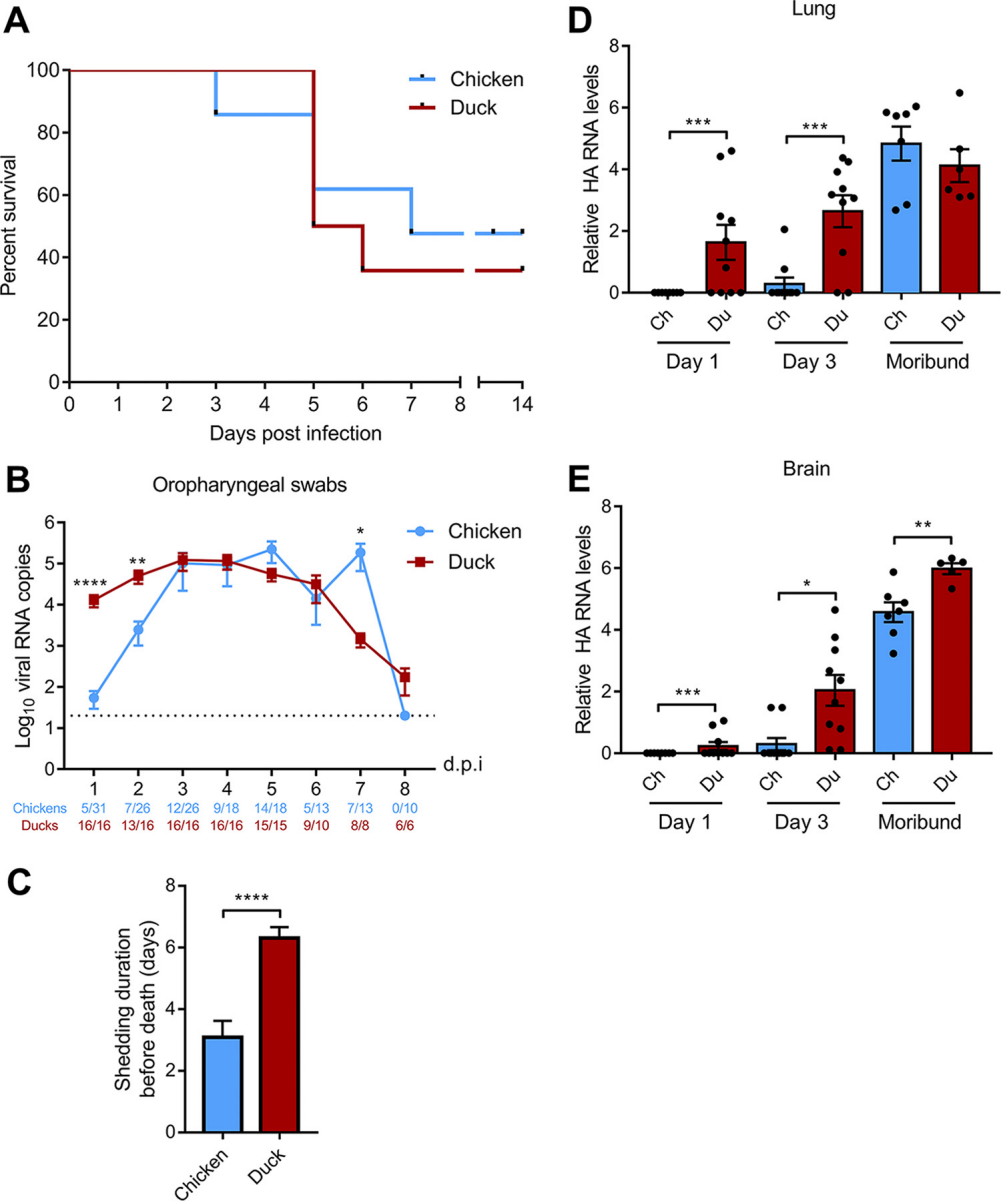
Mortality and viral replication following chicken and duck H5N8 HPAIV infection. (A) Survival curves of H5N8-infected chickens and ducks. (B) Viral shedding was analyzed by quantifying HA RNA levels by RT-qPCR from RNA extracted from oropharyngeal swabs. Statistical analysis was performed with the two-tailed Mann-Whitney test. Results are expressed as means ± SEM. The dotted line represents the limit of detection. (C) Shedding duration, i.e., number of days for which oropharyngeal swabs were positive for HA RNA by RT-qPCR. Statistical analysis was performed with the two-tailed Mann-Whitney test. Results are expressed as means ± SEM. (D and E) Viral loads were analyzed from total RNA extracted from lungs (D) and brain (E). HA RNA levels were normalized using the 2DD-CT method. Ch, chicken; Du: duck; *, *P* < 0.05; **, *P* < 0.01; ***, *P* < 0.001; ****, *P* < 0.0001. Statistical analysis was performed with the two-tailed Mann-Whitney test. Results are expressed as means ± SEM. The dotted line represents the limit of detection. dpi, days postinfection.

Lethality and mean death time were equivalent in ducks and chickens (Fig. 2A). In all four experiments, we observed that all animals displaying clinical signs died, whereas all surviving birds remained clinically healthy throughout the experiments, as described previously (14). Clinical presentations, however, were clearly distinct between ducks and chickens and in were line with previously published field and experimental observations (11, 13–16). Sick chickens displayed dyspnoea, and postmortem examination revealed severe pulmonary congestion and edema, suggesting that chickens died of respiratory dysfunction. On the contrary, sick ducks displayed neurological disorders (including torticollis, tremors of head, ataxia, and convulsions), without any overt respiratory symptoms. Duck postmortem examination revealed pericardial hemorrhages and severe brain congestion.

We measured viral shedding from oropharyngeal swabs by quantifying viral RNA by RT-qPCR. Oropharyngeal shedding was significantly reduced in chickens compared with that in ducks in the first days postinfection (dpi), with a >100-fold difference at 1 dpi (*P* < 0.0001), a >10-fold difference at 2 dpi (*P* < 0.0001), and an 8-fold difference at 3 dpi (*P* < 0.001) (Fig. 2B). Thus, although oropharyngeal viral shedding eventually reached the same level in ducks and chickens, replication appeared to lag in chickens. In accordance with this observation, the shedding duration, i.e., the number of days from the first positive oropharyngeal swab until death, was significantly shorter in chickens compared with that in ducks, indicating that ducks survived longer than chickens during productive viral infection (Fig. 2C).

**FIG 3:**
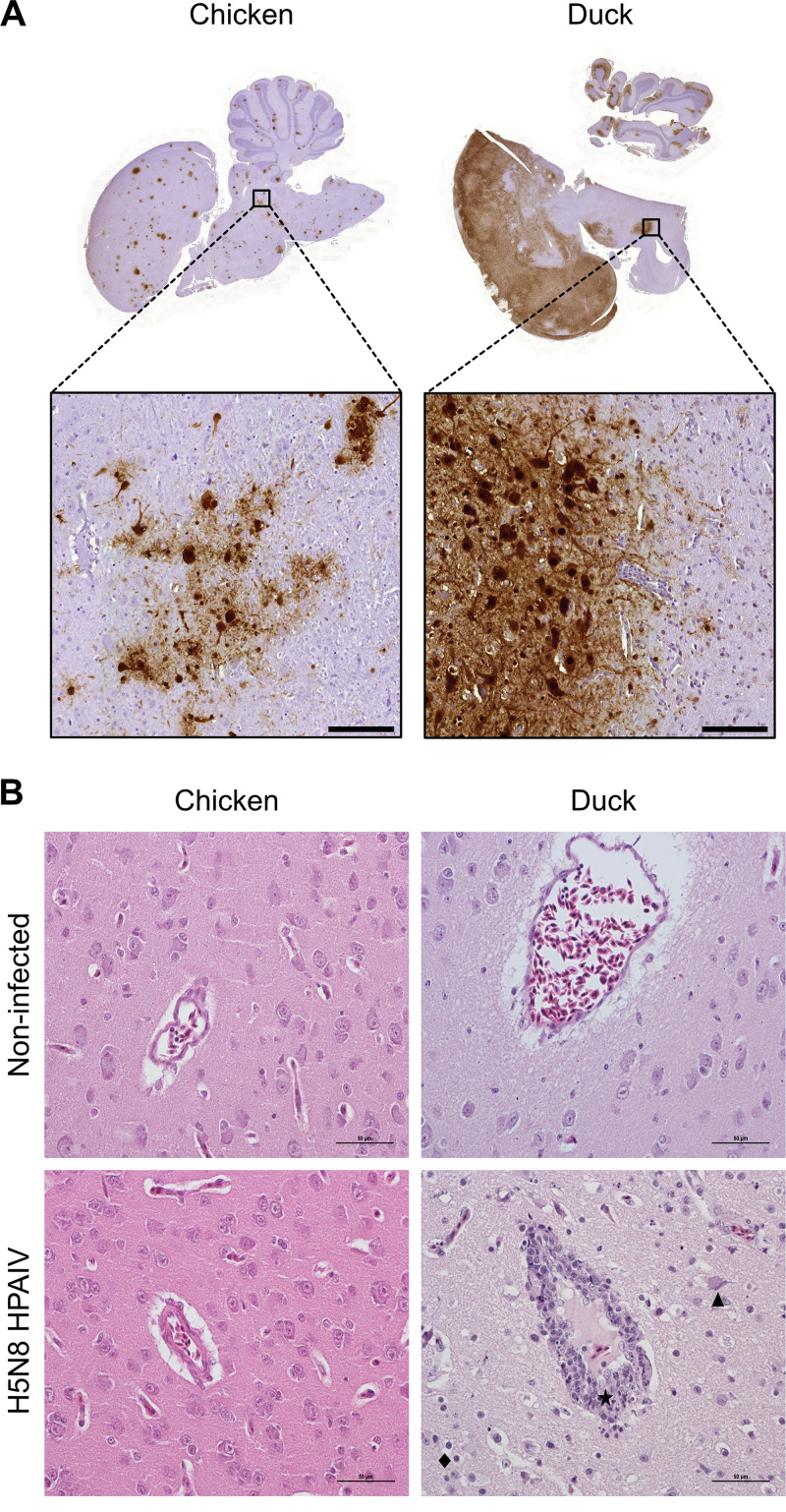
Histopathological analysis of brain samples following H5N8 HPAIV infection. (A) Immunohistochemical anti-NP staining of hematoxylin-counterstained chicken and duck brain sections from moribund animals. Scale bars, 100 μm. (B) Hematoxylin and eosin staining on brain sections from moribund animals. Lymphocytic meningoencephalitis is observed only in the brains of H5N8 HPAIV-infected ducks. The star symbol indicates sites of lymphocytic infiltration of perivascular meningeal spaces, and the triangle indicates neuronal necrosis and the losange focal gliosis. Scale bars, 50 μm.

Necropsies were performed at 1 and 3 dpi and on moribund animals that were euthanized when they reached humane termination criteria. Viral RNA was not detected in the lungs of chickens at 1 dpi, and only 2 chickens out of 10 displayed detectable viral RNA levels in the lungs at 3 dpi (Fig. 2D). In contrast, moribund chickens had high levels of viral RNA in the lungs. These results indicate that H5N8 HPAIV replicated predominantly in the upper respiratory tract of chickens in the early stages of infection and ultimately reached high levels in the lower respiratory tract. In contrast, ducks had significantly higher viral RNA levels in the lungs at 1 and 3 dpi than chickens, which correlated with higher viral RNA levels detected in oropharyngeal swabs at 1 and 2 dpi. We detected significantly higher levels of virus in the duck brains at 1 and 3 dpi and in moribund animals than those in chickens, indicating both earlier and more intense H5N8 HPAIV infection of the central nervous system in ducks than in chickens (Fig. 2E). In line with these results, massive viral antigen staining was detected in the brains of moribund ducks, while antigen staining in the brains of moribund chickens was less pronounced (Fig. 3A).

### Analysis of the host response to H5N8 HPAIV infection in the brain.

To get further insights on the central nervous system lesions caused by H5N8 infection, we performed histopathological analysis of hematoxylin and eosin stain-colored brain sections. Surprisingly, we did not find any histological lesion on chicken brain sections at all time points, even on moribund animals for which viral RNA and viral antigen were detected by RT-qPCR and immunohistochemistry, respectively. In contrast, duck brain tissue was lesioned from 3 dpi. In the duck brain, lesions included lymphocytic meningoencephalitis, neuronal necrosis, neuronophagia, and focal gliosis (Fig. 3B).

**FIG 4:**
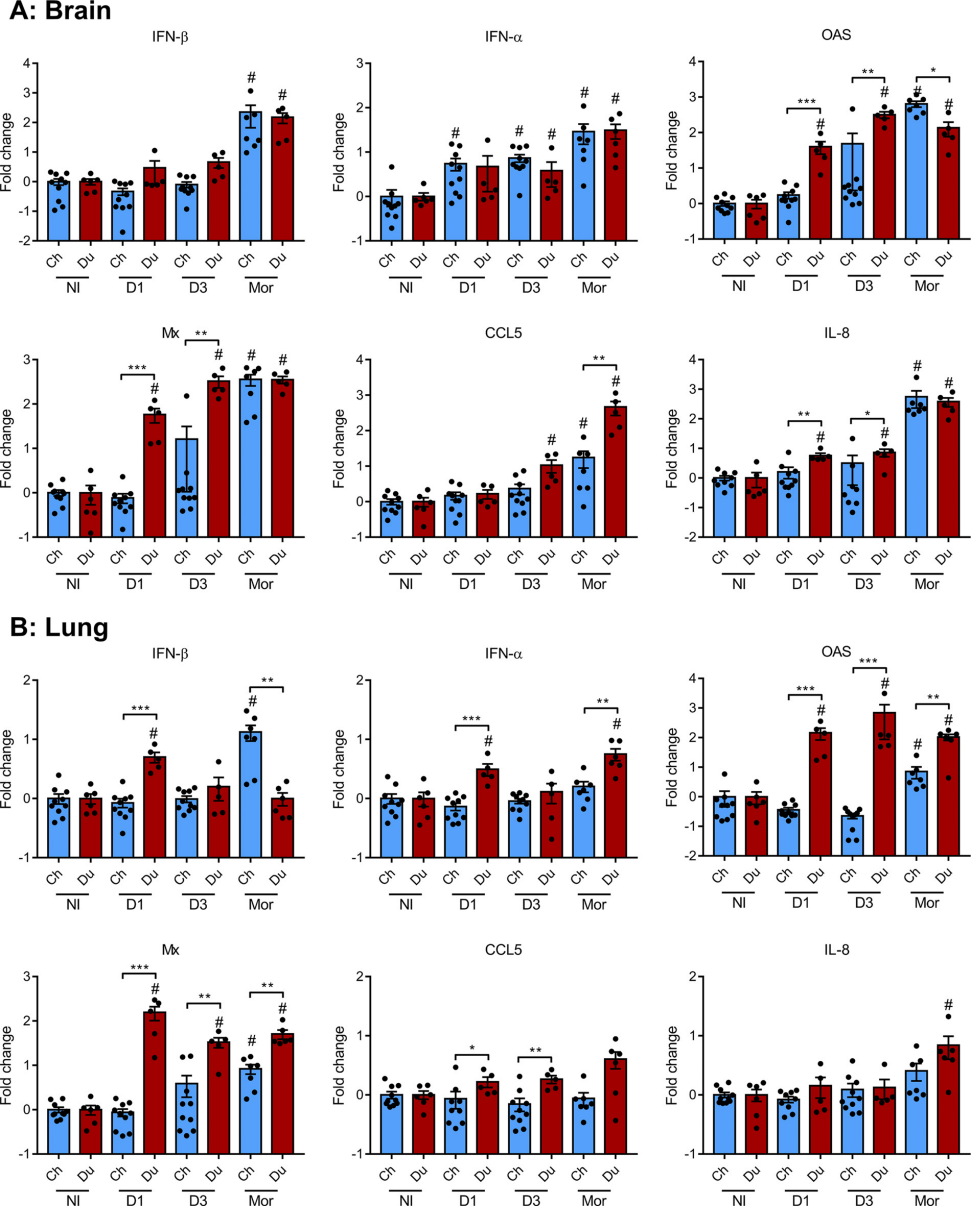
Immune and inflammatory response markers in chicken and duck lungs and brains. mRNA expression levels of IFN-β, IFN-α, OAS, Mx, CCL5, and IL-8 in brain (A) and lung (B) samples, determined by RT-qPCR performed on total RNA. mRNA levels were normalized using the 2DD-CT method and expressed as fold changes over noninfected animals. Statistical analysis was performed using the two-tailed Mann-Whitney test. Results are expressed as means ± SEM. Ch, chicken; Du, duck; D1, day 1; D3, day 3; Mor, moribund; NI, noninfected; #, *P* < 0.05 compared with noninfected animals. *, *P* < 0.05; **, *P* < 0.01; ***, *P* < 0.001.

We evaluated the expression of host immune and inflammatory response markers in the brain using RT-qPCR (Fig. 4A). IFN-β mRNA expression was not upregulated at 1 and 3 dpi. However, we detected a statistically significant upregulation of IFN-β mRNA in the brains of moribund chickens and ducks, without any statistically significant difference between both groups. IFN-α mRNA expression was upregulated at 1 and 3 dpi and in moribund animals, with no statistically significant difference between chickens and ducks. Next, we measured the mRNA levels of the interferon-stimulated genes Mx and OAS, whose expression is a very sensitive indicator of the levels of type I IFN produced locally (17). Mx and OAS mRNA levels were significantly increased in the brains of infected ducks, from 1 dpi onward, and in the brains of moribund chickens. Compared with chickens, duck brain levels of Mx and OAS mRNA levels were significantly increased at 1 dpi and 3 dpi. We detected an upregulation of CCL5 mRNA levels in ducks at 3 dpi and in the brains of moribund chickens. However, CCL5 mRNA levels were significantly higher in the brains of moribund ducks than in the brains of moribund chickens. Brain IL-8 mRNA levels were increased in duck brains from 1 dpi onward, while only moribund chickens displayed increased IL-8 mRNA levels.

In the lungs (Fig. 4B), we detected an upregulation of IFN-β mRNA in ducks at 1 dpi and in moribund chickens. IFN-α mRNA expression was significantly increased in ducks at 1 dpi and in moribund ducks. In line with these observations, in comparison with noninfected animals, the mRNA levels of the interferon-stimulated genes Mx and OAS were significantly increased in the lungs of ducks from 1 dpi onward, whereas they were upregulated only in the lungs of moribund chickens. Importantly, Mx and OAS mRNA levels were significantly higher in the lungs of infected ducks than those in infected chickens from 1 dpi onward. CCL5 mRNAs were modestly upregulated in the lungs of ducks at 1 and 3 dpi, while IL-8 mRNA levels were upregulated only in the lungs of moribund ducks.

**FIG 5:**
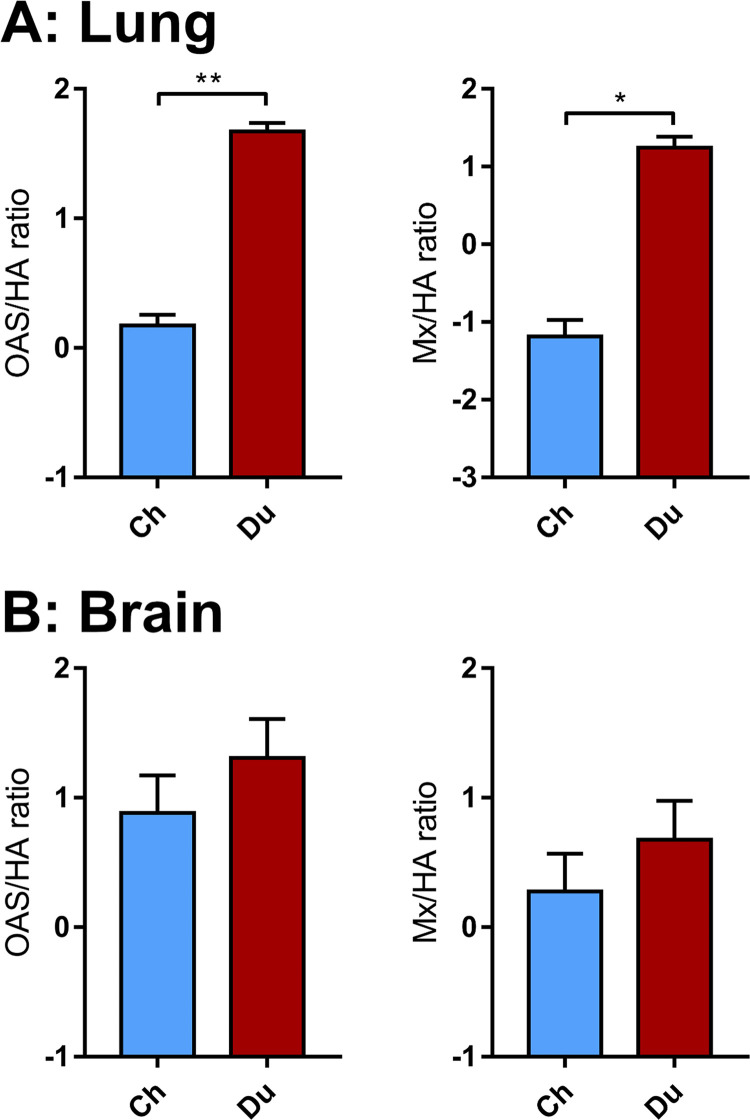
Mean Mx/HA and OAS/HA ratios in the lungs and brain. Mx and OAS mRNA and HA RNA levels from lung (A) and brain (B) samples were normalized using the 2DD-CT method. Statistical analysis used the two-tailed Mann-Whitney test. Results are expressed as means ± SEM. *, *P* < 0.05; **, *P* < 0.01.

Taken together, these results indicate that the innate immune response occurred earlier and was more intense in the brain and lungs of ducks than that in chickens. However, the intensity of the innate immune response depends on the level of viral RNA acting as ligands of the innate immune receptors, as observed previously (14, 18–20). Thus, to further compare the antiviral innate immune response in ducks and chickens, we determined the Mx mRNA/viral RNA and OAS mRNA/viral RNA ratios in the brain and lungs (Fig. 5). These ratios were significantly higher in duck lungs than in chicken lungs, which is in agreement with previous findings (14). Interestingly, ratios in the brain were similar between chickens and ducks. These results suggest that ducks mounted a more efficient innate immune response than chickens in the lungs, while their response was equivalent in the brain. Thus, the higher levels of interferon-stimulated genes Mx and OAS mRNAs observed in the duck brain could be attributed to higher levels of viral RNA, which triggered a more intense innate immune response.

## DISCUSSION

We observed similar levels of viral replication in duck and chicken primary cortical neurons, indicating that duck neurons were not intrinsically more sensitive to H5N8 HPAIV infection than chicken neurons. In addition, the intensity of the innate immune response in the brain was similarly proportional to the viral load in both species, suggesting that the stronger neurotropism observed in duck brains *in vivo* was not due to an impaired central nervous system immune response in ducks compared with that in chickens. Our study suggests that the predominant neurological symptoms could be due to the combination of the early colonization of the central nervous system and of the prolonged survival after the onset of virus replication observed in H5N8 HPAIV-infected ducks. Indeed, shedding duration was significantly shorter in chickens than that in ducks (Fig. 2C). As a consequence, viral replication in the duck central nervous system took place over several days, allowing the virus and the host response to cause significant tissue damage. We hypothesize that chickens died of acute respiratory distress before neurological symptoms could be observed. In accordance with this hypothesis, several reports indicate that birds surviving the peracute phase of HPAIV infections are more likely to develop neurological signs (21–24).

The ability of ducks to survive longer to H5N8 clade 2.3.4.4B HPAIV infection could be due to a more efficient antiviral innate immune response in ducks than that in chickens. Indeed, a substantial body of evidence indicates that ducks mount a more efficient type I interferon-mediated antiviral innate immune response against influenza viruses observed than chickens, possibly because they express a functional RIG-I receptor (19, 25–30). We provide evidence that the antiviral innate immune response to H5N8 HPAIV infection in the duck respiratory tract is significantly more efficient than that in the chicken respiratory tract (Fig. 5A), confirming previously published results (14). We hypothesize that this immune response may allow ducks to better control virus replication in the lungs than chickens and thus allowing sufficient time for viral growth in the central nervous system, whereas chickens succumb to extensive lung damage before lesions accumulate in the central nervous system.

Because of the ability of HPAIV bird infections to spread systemically, HPAIV infection of the central nervous system is not uncommon. HPAIV may enter the central nervous system in birds via the cranial nerves or via hematogenous spread and infection of endothelia, with both routes being strongly favored by the presence of an HA polybasic cleavage site (31–34). Interestingly, the contribution of the HA polybasic cleavage to the infection of the central nervous system in mammals is less clear (35). The presence of a polybasic cleavage site has been proposed as an important determinant for H5N1 HPAIV access to the central nervous system in ferrets (36). In line with these results, there have been several reports of H5N1 HPAIV infection of the central nervous system in humans and other mammals (37–43), including recently described 2.3.4.4B H5N1 HPAIV central nervous system infections in foxes and seals (44, 45). Central nervous system infection has also been described in ferrets infected with a reconstituted 1918 H1N1 virus and in mice infected with the 1918 H1N1-related laboratory strain A/WSN/33 (46–48). Interestingly, although 1918 H1N1 virus and A/WSN/33 lack a polybasic cleavage site, proteolytic activation of their HAs can proceed independently of trypsin-like proteases (49, 50). These observations point to the trypsin-like protease-independent proteolytic activation of HA as a critical determinant of the capacity of influenza A viruses to infect and replicate in the central nervous system of mammals, thus mirroring the well-described mechanisms of central nervous system infection in birds. However, additional viral determinants are likely involved, including the HA sialic acid binding properties, as an H3N2 engineered to express an HA with a polybasic cleavage site failed to infect the central nervous system in ferrets (36, 51). In summary, the cleavability of the HA by trypsin-like protease-independent mechanisms, in association with additional viral determinants, appears as a major determinant of the neurotropism of influenza A in mammals. Importantly, however, central nervous system pathology has also been observed in patients infected with seasonal influenza viruses that are considered nonneurotropic, based on the absence of antigen and viral RNA detection in the central nervous system (52). In the absence of central nervous system infection, the host immune response to viral infection is a likely cause of neurological symptoms, mediated by hyperthermia, circulating cytokines, or activated immune cells, as reviewed in reference 52. Further studies are needed to better understand the determinants leading to central nervous system involvement, especially the potential consequences of the infection of olfactory nerves by respiratory viruses (53, 54).

## MATERIALS AND METHODS

### Viruses.

The HPAIV and LPAIV H5N8 were generated by reverse genetics as described previously, using the eight segments from the A/mallard duck/France/171201g/2017 (H5N8) field isolate. These viruses were referenced by the French biotechnology ethics committee (Haut Conseil des Biotechnologies) and were manipulated exclusively in biosafety level 3 laboratories.

### Animals.

This study was carried out in compliance with European animal welfare regulation. The protocols were approved by the Animal Care and Use Committee (Comité d’Ethique en Science et Santés Animales–115) under protocol 13025-2018012311319709.

Chicken and duck experiments were conducted separately. One-day-old Pekin ducklings (Anas platyrhynchos domesticus, ST5 heavy) were obtained from a commercial hatchery (ORVIA-Couvoir de la Seigneurtière, Vieillevigne, France) and 1-day-old White Leghorn chicks (Gallus gallus domesticus, PA12) from a research hatchery (PFIE, INRAE, Nouzilly, France). Animals were fed *ad libitum* with a starter diet and housed in biosafety level 2 facilities for 2 weeks in a litter-covered floor pen at the National Veterinary School of Toulouse, France. They were then transferred into a biosafety level 3 facility equipped with bird isolators (I-Box; Noroit, Nantes, France) ventilated under negative pressure with HEPA-filtered air. The isolators had a surface area of 2 m^2^; in accordance with French regulation (law NOR AGRG1238753A), a maximum of 8 ducks and 15 chickens were housed in these isolators, to respect a minimum surface area of 0.25 m^2^ per duck and 0.13 m^2^ per chicken.

### *In vivo* infections.

We used samples from two previously published experiments, which we will refer to as duck experiment 1 and chicken experiment 1 (14), as well as samples from two unpublished experiments, which we will refer to as duck experiment 2 and chicken experiment 2.

In all experiments, serum was collected from all animals preinfection to ensure that they were serologically negative to influenza virus by using a commercial influenza A nucleoprotein (NP) antibody competition enzyme-linked immunosorbent assay kit (ID Screen; ID-Vet, Montpellier, France) according to the manufacturer’s instructions. When they were 4 weeks old, animals were infected through the choanal route using an inoculum volume of 100 μL. Noninfected groups received an equivalent volume of allantoic fluid collected from noninfected specific-pathogen-free (SPF) embryonated chicken eggs.

For duck experiment 1, 18 animals were infected with 10^4^ EID_50_ H5N8 HPAIV, while 6 animals were assigned to the noninfected control group. Clinical signs were recorded over 14 days. Oropharyngeal and cloacal swabs were performed on 8 animals daily until 8 dpi. Five animals from each group were euthanized and necropsied at 1 and 3 dpi. Moribund animals reaching humane termination criteria (dyspnea, convulsions, and severe lethargy) were euthanized and also necropsied. For each necropsied animal, brain and lungs were collected and stored in TRIzol reagent (Invitrogen, Carlsbad, CA) or stored in 10% neutral formalin.

For chicken experiment 1, 21 animals were infected with 10^4^ EID_50_ H5N8 HPAIV, while 5 animals were assigned to the noninfected control group. Clinical signs were recorded over 14 days. Oropharyngeal and cloacal swabs were performed on all animals daily until 8 dpi. Five animals from each group were euthanized and necropsied at 1 and 3 dpi. Moribund animals reaching humane termination criteria (dyspnea, convulsions, and severe lethargy) were euthanized and also necropsied. For each necropsied animal, brain and lungs were collected and stored in TRIzol reagent (Invitrogen, Carlsbad, CA) or stored in 10% neutral formalin.

For duck experiment 2, 8 animals were infected with 10^4^ EID_50_ H5N8 HPAIV, while 5 animals were assigned to the noninfected control group. Clinical signs were recorded over 14 days. Oropharyngeal and cloacal swabs were performed on all animals daily until 8 dpi. Only moribund animals were necropsied, and brain and lungs were collected and stored in TRIzol reagent (Invitrogen, Carlsbad, CA) or stored in 10% neutral formalin.

For chicken experiment 2, 20 animals were infected with 10^4^ EID_50_ H5N8 HPAIV, while 5 animals were assigned to the noninfected control group. Clinical signs were recorded over 14 days. Oropharyngeal and cloacal swabs were performed on 10 animals daily until 8 dpi. Five animals from each group were euthanized and necropsied at 1 and 3 dpi. Moribund animals reaching humane termination criteria (dyspnea, convulsions, and severe lethargy) were euthanized and also necropsied. For each necropsied animal, brain and lungs were collected and stored in TRIzol reagent (Invitrogen, Carlsbad, CA) or stored in 10% neutral formalin.

### Viral quantification from oropharyngeal swabs.

Oropharyngeal swabs were briefly vortexed in 500 μL of sterile phosphate-buffered saline (PBS) and H5N8 RNA was extracted from 200 μL using a QiaCube automated platform according to the manufacturer’s instructions (Cador Pathogen QIAcube HT kit; Qiagen, Toronto, Canada). Influenza nucleic acid load was determined by RT-qPCR using primers targeting HA. RT-qPCR was performed in 384-well plates in a final volume of 10 μL using the Bravo automated liquid handling platform (Agilent Technologies, Palo Alto, CA) and a ViaaA 7 real-time PCR system (Applied Biosystems) at the GeT-TRiX platform (GénoToul, Génopole, Toulouse, France). Mixes were prepared according to the manufacturer’s instructions (iTaq SYBR green one-step; Bio-Rad) with 2 μL of RNA and a final concentration of 0.5 μM of each primer (Table 1). Absolute quantification was performed using a standard curve based on 10-fold serial dilutions (from 10^1^ to 10^7^ copies) of plasmid containing the H5N8 HA gene.

**TABLE 1:** Primers used for qPCR^*a*^

Gene name	Primer sequence direction	Primer sequence (5′–3′)	NCBI accession no. or reference
HA H5N8 HPAIV	F	GACCTCTGTTACCCAGGGAGCCT	14
	R	GGACAAGCTGCGCTTACCCCT	
Chicken GADPH	F	TCCTCTCTGGCAAAGTCCAAG	14
	R	CACAACATACTCAGCACCTGC	
Duck GAPDH	F	CCACTTCCGGGGCACTGTCA	56
	R	AGCACCAGCATCTGCCCACT	
Chicken IFN-α	F	AATGCTTGGACAGCAGCGAC	14
	R	TTGTCTTGGAGGAAGGTGTG	
Duck IFN-α	F	CAACGACACGCAGCAAGC	14
	R	GGGTGTCGAAGAGGTGTTGG	
Chicken IFN-β	F	GCTTCGTAAACCAAGGCACG	14
	R	GAGCTCGACTTTTCATCCATTG	
Duck IFN-β	F	TCTACAGAGCCTTGCCTGCAT	56
	R	TGTCGGTGTCCAAAAGGATGT	
Chicken Mx	F	CACTGCAACAAGCAAAGAAGGA	14
	R	TGATCAACCCCACAAGGAAAA	
Duck Mx	F	TCACACGAAGGCCTATTTTACTGG	14
	R	GTCGCCGAAGTCATGAAGGA	
Chicken OAS	F	CGCTCCCTCAGCCTCACCCT	NM_001397447
	R	CCGGGCGGGCATCCTGAATC	
Duck OAS	F	CCGCCAAGCTGAAGAACCTG	56
	R	CGCCCTGCTCCCAGGTATAG	
Chicken CCL5	F	CCCTCTCCATCCTCCTGGTT	57
	R	TATCAGCCCCAAACGGAGAT	
Duck CCL5	F	CACCAGCAGCAAATGCCCACAGC	58
	R	CAAGCAGGATTTCTGGTCCATGCC	
Chicken IL-8	F	CTGCGGTGCCAGTGCATTAG	AJ009800
	R	AGCACACCTCTCTTCCATCC	
Duck IL-8	F	AGGACAACAGAGAGGTGTGCTTG	NM_001310420
	R	GCCTTTACGATCCGCTGTACC	

a CCL5, chemokine (C-C motif) ligand 5; GAPDH, glyceraldehyde-3-phosphate dehydrogenase; IFN-α, interferon alpha; IFN-β, interferon beta; IL-8, interleukin-8; Mx, myxovirus resistance dynamin like GTPase; OAS, oligoadenylate synthase; F, forward; R, reverse.

### RNA extraction from tissue samples and cDNA synthesis.

A total of 30-mg portions of tissue were placed in tubes with beads (Precellys lysis kit; Stretton Scientific, Ltd., Stretton, UK) filled with 1 mL of TRIzol reagent (Invitrogen, Carlsbad, CA) and mixed for 5 s at 6,000 rpm three times in a bead beater (Precellys 24; Bertin Technologies, Montigny-le-Bretonneux, France). After the TRIzol extraction, the aqueous phase was transferred to an RNA extraction column and processed according to the manufacturer’s instructions (RNeasy minikit; Qiagen, Toronto, Canada). RNA was then treated with DNase (RNase I; Invitrogen, Thermo Fisher Scientific) to remove genomic DNA contamination. cDNA was synthesized by reverse transcription of 500 ng of total RNA using oligo(dT)18 (0.25 μg) and random hexamer (0.1 μg) primers and a RevertAid first-strand cDNA synthesis kit (Invitrogen, Thermo Fisher Scientific) according to the manufacturer’s instructions.

### Quantitative PCR from tissue samples.

Quantitative PCR for the analysis of host gene expression was performed in 384-well plates in a final volume of 10 μL using a Bravo automated liquid handling platform (Agilent Technologies, Palo Alto, CA) and a ViiA 7 real-time PCR system (Applied Biosystems, Foster City, CA) at the GeT-TRiX platform (GenoToul, Genopole, Toulouse, France). Mixes were prepared according to the manufacturer’s instructions (iTaq SYBR green PCR; Bio-Rad) with 2 μL of 1:20 diluted cDNA and a final 0.5 μM concentration of each primer. Quantification of influenza virus nucleic acid load in tissues was performed in 96-well plates with a 10-μL final volume according to the manufacturer’s instructions (iTaq SYBR green PCR; Bio-Rad), along with 2 μL of cDNA and a final 0.5 μM concentration of HA-specific primers. qPCR was performed on a LightCycler 96 instrument (Roche). Relative quantification was carried out by using the threshold cycle (2^−ΔΔ^*^CT^*) method. RNA levels were normalized with GAPDH mRNA levels. Primer sequences are described in Table 1.

### Histopathological examination.

All animals were subjected to a complete postmortem examination. Tissue samples were taken and stored in 10% neutral formalin. After fixation, tissues were processed in paraffin blocks, sectioned at 4 μm, and stained with hematoxylin and eosin for microscopic examination. A board-certified veterinary pathologist who was blind to the experimental conditions assessed lesions histologically.

### Immunohistochemistry.

Brains were taken and stored in 10% neutral formalin. After fixation, tissues were processed in paraffin blocks and sectioned at 4 μm, and immunohistochemistry was performed on paraffin-embedded sections with a monoclonal mouse anti-nucleoprotein influenza A virus antibody (Argene; 11-030; pronase 0.05% retrieval solution, 10 min at 37°C, antibody dilution of 1/50, incubation overnight at 4°C). The immunohistochemical staining was revealed with a biotinylated polyclonal goat anti-mouse immunoglobulin conjugated with horseradish peroxidase (HRP; Dako; LSAB2 system-HRP, K0675) and the diaminobenzidine HRP chromogen (Thermo Scientific; TA-125-HDX). Negative controls comprised sections incubated either without specific primary antibody or with another monoclonal antibody of the same isotype (IgG2).

### Chicken and duck primary cortical neuron culture.

SPF White Leghorn (PA12) embryonated chicken eggs (PFIE, INRAE, Nouzilly, France) and Pekin duck (ST5 Heavy) embryonated eggs (ORVIA-Couvoir de la Seigneurtière, Vieillevigne, France) were incubated for 13 and 14 days, respectively, at 37°C to account for equivalent stages of development (55). After brain dissection, cortexes were isolated and the meninges and hippocampus were removed in cold PBS containing 1 g/L glucose and 1% antibiotics (penicillin-streptomycin). Cortex tissue was dissociated by a 15-min incubation at 37°C in PBS containing 10 U/mL papain (Worthington Biochemical, Lakewood, NJ) followed by a gentle dissociation in Low-Ovomucoid buffer containing PBS 1 × 1.5 mg/mL bovine serum albumin (BSA), 1.5 mg/mL trypsin inhibitor from chicken egg white (Sigma-Aldrich), and 66.7 μg/mL DNase I from bovine pancreas (Roche). After a prefiltration step on a 100-μm nylon cell strainer, neurons were centrifuged through a 4% BSA cushion in Neurobasal medium (Gibco), counted, and then plated on variable supports (glass coverslips in 24-well or 4-well plates, 6-well and 12-well culture plates depending on the experiments). Supports were previously overnight coated with 500 μg/mL polyDL-ornithine diluted in sterile distilled water (Sigma-Aldrich) and then coated for 2 h with 5 μg/mL Laminin from Engelbreth-Holm-Swarm murine sarcoma basement membrane (Sigma-Aldrich) diluted in Neurobasal medium (Roche). Neuron cultures were maintained in complete neuronal culture medium composed of serum-free Neurobasal medium supplemented with 2 mM glutamine (Gibco), 1% penicillin-streptomycin, and 2% B-27-plus supplement (Gibco). For immunofluorescent staining, neurons were seeded on coated glass coverslips into 24-wells plates. For viral replication kinetics assays and host response analysis, neurons were seeded in 12-well plates. All the analyses were initiated on neurons that had been cultured for 4 days at 37°C with 5% CO_2_.

### Primary neuron infection with HPAIV or LPAIV H5N8.

Infections with HPAIV were carried out in Neurobasal medium supplemented with 2 mM glutamine (Gibco), 1% penicillin-streptomycin, and 2% B-27-plus supplement (Gibco). As the B-27-plus supplement was found to inhibit exogenous trypsin, comparisons of HPAIV and LPAIV replication were carried out in Opti-MEM medium (Gibco) instead of in Neurobasal medium and B-27-plus. For immunofluorescent staining, neurons were fixed at 8 h postinfection. For host response analyses, cells were harvested 24 h postinfection.

### Immunofluorescence.

Infected neurons were fixed for 15 min at room temperature with PBS containing 4% paraformaldehyde (PFA; Electron Microscopy Sciences, Hatfield, England), washed twice in PBS, and then permeabilized using PBS plus 0.1% Triton X-100 for 10 min. Coverslips were rinsed with PBS and blocked for 1 h with PBS containing 5% BSA (blocking buffer) at room temperature. To assess neuronal integrity, we used an avian neuron-specific beta-III tubulin antibody (Monoclonal Mouse IgG 2a from Biotechne) diluted at 1:400. To control infection, we used a rabbit-monoclonal anti-influenza A virus nonstructural protein 1 (kindly provided by Daniel Marc from INRAE Nouzilly, France) diluted at 1:400. Upon incubation with primary antibodies, neurons were washed 3 times with PBS-0.1% Triton X-100 and incubated with a secondary goat anti-mouse antibody coupled to Alexa Fluor 594 (Invitrogen) diluted at 1:2,000 and secondary donkey anti-rabbit antibody coupled to Alexa Fluor 488 diluted at 1:1,000, for 1 h at room temperature. After 4 washes in PBS-0.1% Triton X-100 and one wash with distilled water, coverslips were placed on glass microscopy slides with mounting medium Vectashield with 4′,6-diamidino-2-phenylindole (DAPI; (Eurobio). Fluorescence-based measurements were performed on Leica SP8 STED inverted confocal microscopes with a 63× objective on a TRI-Genotoul Infinity cell imaging facility platform (INSERM, Toulouse, France). Image-based analysis of the purity of cortical neuron cultures was performed using ImageJ software from two independent experiments in ducks and two independent experiments in chickens. For each experiment, the percentage of cortical neurons was evaluated from 10 randomly selected microscopic fields by calculating the number of beta-III-tubulin-stained neurons/total number of DAPI-stained nuclei.

### Reverse transcription and qPCR from neuron samples.

For viral replication kinetics, viral RNA extraction was performed on 140-μL supernatant collected at 1, 24, 48, and 72 h postinfection according to the manufacturer’s instructions (QIAamp viral RNA; Qiagen, Toronto, Canada). Influenza nucleic acid load was determined by RT-qPCR using primers targeting the HA gene in a final volume of 10 μL. Mixes were prepared according to the manufacturer’s instructions (iTaq Universal SYBR green one-step kit; Bio-Rad) with 2 μL of RNA and a final concentration of 0.3 μM of each primer. Absolute quantification was performed using a standard curve based on 10-fold serial dilutions (from 10^1^ to 10^7^ copies) of plasmid containing thevH5N8 HA gene. For host response analysis, the supernatant was removed at 24 h postinfection. Cells were scratched and total RNA was extracted according to the manufacturer’s instructions (RNeasy minikit; Qiagen, Toronto, Canada) followed by DNase treatment according to the manufacturer’s instructions (DNase I; Thermo Fisher Scientific). cDNA was synthesized by reverse transcription of 500 ng of total RNA using either both oligo(dT)18 (0.25 μg) and random hexamer (0.1 μg) primers and a RevertAid first-strand cDNA synthesis kit (Invitrogen, Thermo Fisher Scientific) according to the manufacturer’s instructions. Quantitative PCR for the analysis of host gene expression was performed in 384-well plates in a final volume of 10 μL using a Bravo automated liquid handling platform (Agilent Technologies, Palo Alto, CA) and a ViiA 7 real-time PCR system (Applied Biosystems, Foster City, CA) at the GeT-TRiX platform (GenoToul, Genopole, Toulouse, France). Mixes were prepared according to the manufacturer’s instructions (iTaq SYBR green PCR, Bio-Rad) with 2 μL of 1:20 diluted cDNA and a final 0.5 μM concentration of each primer (Table 1).

### Data availability.

Segment sequences can be found on GenBank (accession numbers MK859904 to MK859911 and MK859926 to MK859933).

## References

[1] Lee D-H, Torchetti MK, Winker K, Ip HS, Song C-S, Swayne DE. 2015. Intercontinental spread of Asian-origin H5N8 to North America through Beringia by migratory birds. J Virol 89:6521–6524. doi:10.1128/JVI.00728-15. 25855748 PMC4474297

[2] The Global Consortium For H5n8 and Related Influenza Viruses. 2016. Role for migratory wild birds in the global spread of avian influenza H5N8. Science 354:213–217. doi:10.1126/science.aaf8852. 27738169 PMC5972003

[3] Pohlmann A, King J, Fusaro A, Zecchin B, Banyard AC, Brown IH, Byrne AMP, Beerens N, Liang Y, Heutink R, Harders F, James J, Reid SM, Hansen RDE, Lewis NS, Hjulsager C, Larsen LE, Zohari S, Anderson K, Bröjer C, Nagy A, Savič V, van Borm S, Steensels M, Briand F-X, Swieton E, Smietanka K, Grund C, Beer M, Harder T. 2022. Has epizootic become enzootic? Evidence for a fundamental change in the infection dynamics of highly pathogenic avian influenza in Europe, 2021. mBio 13:e0060922. doi:10.1128/mbio.00609-22. 35726917 PMC9426456

[4] Yamaji R, Saad MD, Davis CT, Swayne DE, Wang D, Wong FYK, McCauley JW, Peiris JSM, Webby RJ, Fouchier RAM, Kawaoka Y, Zhang W. 2020. Pandemic potential of highly pathogenic avian influenza clade 2.3.4.4 A(H5) viruses. Rev Med Virol 30:e2099. doi:10.1002/rmv.2099. 32135031 PMC9285678

[5] Pyankova OG, Susloparov IM, Moiseeva AA, Kolosova NP, Onkhonova GS, Danilenko AV, Vakalova EV, Shendo GL, Nekeshina NN, Noskova LN, Demina JV, Frolova NV, Gavrilova EV, Maksyutov RA, Ryzhikov AB. 2021. Isolation of clade 2.3.4.4b A(H5N8), a highly pathogenic avian influenza virus, from a worker during an outbreak on a poultry farm, Russia, December 2020. Eurosurveillance 26:2100439. doi:10.2807/1560-7917.ES.2021.26.24.2100439. 34142650 PMC8212591

[6] Böttcher-Friebertshäuser E, Garten W, Matrosovich M, Klenk HD. 2014. The hemagglutinin: a determinant of pathogenicity, p 3–34. *In* Compans RW, Oldstone MBA (ed). Influenza Pathogenesis and Control—Volume I. Springer International Publishing, Switzerland. 10.1007/82_2014_384 25031010

[7] Lee D-H, Criado MF, Swayne DE. 2021. Pathobiological origins and evolutionary history of highly pathogenic avian influenza viruses. Cold Spring Harb Perspect Med 11:a038679. doi:10.1101/cshperspect.a038679. 31964650 PMC7849344

[8] EFSA Panel on Animal Health and Welfare (AHAW), More S, Bicout D, Bøtner A, Butterworth A, Calistri P, Depner K, Edwards S, Garin-Bastuji B, Good M, Gortázar Schmidt C, Michel V, Miranda MA, Nielsen SS, Raj M, Sihvonen L, Spoolder H, Thulke H-H, Velarde A, Willeberg P, Winckler C, Breed A, Brouwer A, Guillemain M, Harder T, Monne I, Roberts H, Baldinelli F, Barrucci F, Fabris C, Martino L, Mosbach-Schulz O, Verdonck F, Morgado J, Stegeman JA. 2017. Avian influenza. EFSA J 15:e04991. doi:10.2903/j.efsa.2017.4991. 32625288 PMC7009867

[9] Keawcharoen J, van Riel D, van Amerongen G, Bestebroer T, Beyer WE, van Lavieren R, Osterhaus ADME, Fouchier RAM, Kuiken T. 2008. Wild ducks as long-distance vectors of highly pathogenic avian influenza virus (H5N1). Emerg Infect Dis 14:600–607. doi:10.3201/eid1404.071016. 18394278 PMC2570914

[10] Beerens N, Germeraad EA, Venema S, Verheij E, Pritz-Verschuren SBE, Gonzales JL. 2021. Comparative pathogenicity and environmental transmission of recent highly pathogenic avian influenza H5 viruses. Emerg Microbes Infect 10:97–108. doi:10.1080/22221751.2020.1868274. 33350337 PMC7832006

[11] Kleyheeg E, Slaterus R, Bodewes R, Rijks JM, Spierenburg MAH, Beerens N, Kelder L, Poen MJ, Stegeman JA, Fouchier RAM, Kuiken T, van der Jeugd HP. 2017. Deaths among wild birds during highly pathogenic avian influenza A(H5N8) virus outbreak, the Netherlands. Emerg Infect Dis 23:2050–2054. doi:10.3201/eid2312.171086. 29148372 PMC5708256

[12] Lee D-H, Bertran K, Kwon J-H, Swayne DE. 2017. Evolution, global spread, and pathogenicity of highly pathogenic avian influenza H5Nx clade 2.3.4.4. J Vet Sci 18:269–280. doi:10.4142/jvs.2017.18.S1.269. 28859267 PMC5583414

[13] Grund C, Hoffmann D, Ulrich R, Naguib M, Schinköthe J, Hoffmann B, Harder T, Saenger S, Zscheppang K, Tönnies M, Hippenstiel S, Hocke A, Wolff T, Beer M. 2018. A novel European H5N8 influenza A virus has increased virulence in ducks but low zoonotic potential. Emerg Microbes Infect 7:132. doi:10.1038/s41426-018-0130-1. 30026505 PMC6053424

[14] Bessière P, Figueroa T, Coggon A, Foret-Lucas C, Houffschmitt A, Fusade-Boyer M, Dupré G, Guérin J-L, Delverdier M, Volmer R. 2022. Opposite outcomes of the within-host competition between highly and low pathogenic H5N8 avian influenza viruses in chickens compared to ducks. J Virol 96:e01366-21. doi:10.1128/JVI.01366-21. 34613804 PMC8754203

[15] Schreuder J, Manders TTM, Elbers ARW, van der Spek AN, Bouwstra RJ, Stegeman JA, Velkers FC. 2021. Highly pathogenic avian influenza subtype H5Nx clade 2.3.4.4 outbreaks in Dutch poultry farms, 2014–2018: clinical signs and mortality. Transbound Emerg Dis 68:88–97. doi:10.1111/tbed.13597. 32418364 PMC8048556

[16] Bányai K, Bistyák AT, Thuma Á, Gyuris É, Ursu K, Marton S, Farkas SL, Hortobágyi E, Bacsadi Á, Dán Á. 2016. Neuroinvasive influenza virus A(H5N8) in fattening ducks, Hungary, 2015. Infect Genet Evol 43:418–423. doi:10.1016/j.meegid.2016.05.027. 27215706

[17] Holzinger D, Jorns C, Stertz S, Boisson-Dupuis S, Thimme R, Weidmann M, Casanova J-L, Haller O, Kochs G. 2007. Induction of MxA gene expression by influenza A virus requires type I or type III interferon signaling. J Virol 81:7776–7785. doi:10.1128/JVI.00546-06. 17494065 PMC1933351

[18] Baccam P, Beauchemin C, Macken CA, Hayden FG, Perelson AS. 2006. Kinetics of influenza A virus infection in humans. J Virol 80:7590–7599. doi:10.1128/JVI.01623-05. 16840338 PMC1563736

[19] Soubies SM, Hoffmann TW, Croville G, Larcher T, Ledevin M, Soubieux D, Quéré P, Guérin J-L, Marc D, Volmer R. 2013. Deletion of the C-terminal ESEV domain of NS1 does not affect the replication of a low-pathogenic avian influenza virus H7N1 in ducks and chickens. J Gen Virol 94:50–58. doi:10.1099/vir.0.045153-0. 23052391

[20] Volmer C, Soubies SM, Grenier B, Guérin J-L, Volmer R. 2011. Immune response in the duck intestine following infection with low-pathogenic avian influenza viruses or stimulation with a Toll-like receptor 7 agonist administered orally. J Gen Virol 92:534–543. doi:10.1099/vir.0.026443-0. 21123544

[21] Perkins LEL, Swayne DE. 2003. Comparative susceptibility of selected avian and mammalian species to a Hong Kong-origin H5N1 high-pathogenicity avian influenza virus. Avian Dis 47:956–967. doi:10.1637/0005-2086-47.s3.956. 14575094

[22] Swayne DE. 2007. Understanding the complex pathobiology of high pathogenicity avian influenza viruses in birds. Avian Dis 51:242–249. doi:10.1637/7763-110706-REGR.1. 17494560

[23] Klopfleisch R, Werner O, Mundt E, Harder T, Teifke JP. 2006. Neurotropism of highly pathogenic avian influenza virus A/chicken/indonesia/2003 (H5N1) in experimentally infected pigeons (Columbia livia f. domestica). Vet Pathol 43:463–470. doi:10.1354/vp.43-4-463. 16846988

[24] Leyson C, Youk S-S, Smith D, Dimitrov K, Lee D-H, Larsen LE, Swayne DE, Pantin-Jackwood MJ. 2019. Pathogenicity and genomic changes of a 2016 European H5N8 highly pathogenic avian influenza virus (clade 2.3.4.4) in experimentally infected mallards and chickens. Virology 537:172–185. doi:10.1016/j.virol.2019.08.020. 31493656 PMC6901708

[25] Kuchipudi SV, Tellabati M, Sebastian S, Londt BZ, Jansen C, Vervelde L, Brookes SM, Brown IH, Dunham SP, Chang K-C. 2014. Highly pathogenic avian influenza virus infection in chickens but not ducks is associated with elevated host immune and pro-inflammatory responses. Vet Res 45:118. doi:10.1186/s13567-014-0118-3. 25431115 PMC4246556

[26] Cornelissen JBWJ, Vervelde L, Post J, Rebel JMJ. 2013. Differences in highly pathogenic avian influenza viral pathogenesis and associated early inflammatory response in chickens and ducks. Avian Pathol 42:347–364. doi:10.1080/03079457.2013.807325. 23782222

[27] Cornelissen JBWJ, Post J, Peeters B, Vervelde L, Rebel JMJ. 2012. Differential innate responses of chickens and ducks to low-pathogenic avian influenza. Avian Pathol 41:519–529. doi:10.1080/03079457.2012.732691. 23237364

[28] Burggraaf S, Karpala AJ, Bingham J, Lowther S, Selleck P, Kimpton W, Bean AGD. 2014. H5N1 infection causes rapid mortality and high cytokine levels in chickens compared to ducks. Virus Res 185:23–31. doi:10.1016/j.virusres.2014.03.012. 24657784 PMC7127704

[29] Barber MRW, Aldridge JR, Webster RG, Magor KE. 2010. Association of RIG-I with innate immunity of ducks to influenza. Proc Natl Acad Sci USA 107:5913–5918. doi:10.1073/pnas.1001755107. 20308570 PMC2851864

[30] Magor KE, Miranzo Navarro D, Barber MRW, Petkau K, Fleming-Canepa X, Blyth GAD, Blaine AH. 2013. Defense genes missing from the flight division. Dev Comp Immunol 41:377–388. doi:10.1016/j.dci.2013.04.010. 23624185 PMC7172724

[31] Kobayashi Y, Horimoto T, Kawaoka Y, Alexander DJ, Itakura C. 1996. Neuropathological studies of chickens infected with highly pathogenic avian influenza viruses. J Comp Pathol 114:131–147. doi:10.1016/s0021-9975(96)80003-x. 8920214

[32] Pantin-Jackwook MJ, Swayne DE. 2009. Pathogenesis and pathobiology of avian influenza virus infection in birds. Rev Sci Tech 28:113–136. doi:10.20506/rst.28.1.1869. 19618622

[33] Chaves AJ, Busquets N, Valle R, Rivas R, Vergara-Alert J, Dolz R, Ramis A, Darji A, Majó N. 2011. Neuropathogenesis of a highly pathogenic avian influenza virus (H7N1) in experimentally infected chickens. Vet Res 42:106. doi:10.1186/1297-9716-42-106. 21982125 PMC3199250

[34] Chaves AJ, Vergara-Alert J, Busquets N, Valle R, Rivas R, Ramis A, Darji A, Majó N. 2014. Neuroinvasion of the highly pathogenic influenza virus H7N1 is caused by disruption of the blood brain barrier in an avian model. PLoS One 9:e115138. doi:10.1371/journal.pone.0115138. 25506836 PMC4266681

[35] Veldhuis Kroeze E, Bauer L, Caliendo V, van Riel D. 2021. In vivo models to study the pathogenesis of extra-respiratory complications of influenza A virus infection. Viruses 13:848. doi:10.3390/v13050848. 34066589 PMC8148586

[36] Schrauwen EJA, Herfst S, Leijten LM, van Run P, Bestebroer TM, Linster M, Bodewes R, Kreijtz JHCM, Rimmelzwaan GF, Osterhaus ADME, Fouchier RAM, Kuiken T, van Riel D. 2012. The multibasic cleavage site in H5N1 virus is critical for systemic spread along the olfactory and hematogenous routes in ferrets. J Virol 86:3975–3984. doi:10.1128/JVI.06828-11. 22278228 PMC3302532

[37] de Jong MD, Bach VC, Phan TQ, Vo MH, Tran TT, Nguyen BH, Beld M, Le TP, Truong HK, Nguyen VVC, Tran TH, Do QH, Farrar J. 2005. Fatal avian influenza A (H5N1) in a child presenting with diarrhea followed by coma. N Engl J Med 352:686–691. doi:10.1056/NEJMoa044307. 15716562

[38] Thanawongnuwech R, Amonsin A, Tantilertcharoen R, Damrongwatanapokin S, Theamboonlers A, Payungporn S, Nanthapornphiphat K, Ratanamungklanon S, Tunak E, Songserm T, Vivatthanavanich V, Lekdumrongsak T, Kesdangsakonwut S, Tunhikorn S, Poovorawan Y. 2005. Probable tiger-to-tiger transmission of avian influenza H5N1. Emerg Infect Dis 11:699–701. doi:10.3201/eid1105.050007. 15890122 PMC3320363

[39] Keawcharoen J, Oraveerakul K, Kuiken T, Fouchier RAM, Amonsin A, Payungporn S, Noppornpanth S, Wattanodorn S, Theambooniers A, Tantilertcharoen R, Pattanarangsan R, Arya N, Ratanakorn P, Osterhaus DME, Poovorawan Y. 2004. Avian influenza H5N1 in tigers and leopards. Emerg Infect Dis 10:2189–2191. doi:10.3201/eid1012.040759. 15663858 PMC3323383

[40] Rimmelzwaan GF, van Riel D, Baars M, Bestebroer TM, van Amerongen G, Fouchier RAM, Osterhaus ADME, Kuiken T. 2006. Influenza A virus (H5N1) infection in cats causes systemic disease with potential novel routes of virus spread within and between hosts. Am J Pathol 168:176–183. doi:10.2353/ajpath.2006.050466. 16400021 PMC1592682

[41] Klopfleisch R, Wolf PU, Wolf C, Harder T, Starick E, Niebuhr M, Mettenleiter TC, Teifke JP. 2007. Encephalitis in a stone marten (Martes foina) after natural infection with highly pathogenic avian influenza virus subtype H5N1. J Comp Pathol 137:155–159. doi:10.1016/j.jcpa.2007.06.001. 17689552

[42] Shinya K, Makino A, Hatta M, Watanabe S, Kim JH, Hatta Y, Gao P, Ozawa M, Le QM, Kawaoka Y. 2011. Subclinical brain injury caused by H5N1 influenza virus infection. J Virol 85:5202–5207. doi:10.1128/JVI.00239-11. 21389133 PMC3126180

[43] Mak GCK, Kwan MY-W, Mok CKP, Lo JYC, Peiris M, Leung CW. 2018. Influenza A(H5N1) virus infection in a child with encephalitis complicated by obstructive hydrocephalus. Clin Infect Dis 66:136–139. doi:10.1093/cid/cix707. 29020163 PMC5850530

[44] Floyd T, Banyard AC, Lean FZX, Byrne AMP, Fullick E, Whittard E, Mollett BC, Bexton S, Swinson V, Macrelli M, Lewis NS, Reid SM, Núñez A, Duff JP, Hansen R, Brown IH. 2021. Encephalitis and death in wild mammals at a rehabilitation center after infection with highly pathogenic avian influenza A(H5N8) virus, United Kingdom. Emerg Infect Dis 27:2856–2863. doi:10.3201/eid2711.211225. 34670647 PMC8544989

[45] Rijks JM, Hesselink H, Lollinga P, Wesselman R, Prins P, Weesendorp E, Engelsma M, Heutink R, Harders F, Kik M, Rozendaal H, van den Kerkhof H, Beerens N. 2021. Highly pathogenic avian influenza A(H5N1) virus in wild red foxes, the Netherlands, 2021. Emerg Infect Dis 27:2960–2962. doi:10.3201/eid2711.211281. 34670656 PMC8544991

[46] de Wit E, Siegers JY, Cronin JM, Weatherman S, van den Brand JM, Leijten LM, van Run P, Begeman L, van den Ham H-J, Andeweg AC, Bushmaker T, Scott DP, Saturday G, Munster VJ, Feldmann H, van Riel D. 2018. 1918 H1N1 influenza virus replicates and induces proinflammatory cytokine responses in extrarespiratory tissues of ferrets. J Infect Dis 217:1237–1246. doi:10.1093/infdis/jiy003. 29329410 PMC6018876

[47] Takahashi M, Yamada T, Nakajima S, Nakajima K, Yamamoto T, Okada H. 1995. The substantia nigra is a major target for neurovirulent influenza A virus. J Exp Med 181:2161–2169. doi:10.1084/jem.181.6.2161. 7760004 PMC2192055

[48] Aronsson F, Robertson B, Ljunggren H-G, Kristensson K. 2003. Invasion and persistence of the neuroadapted influenza virus A/WSN/33 in the mouse olfactory system. Viral Immunol 16:415–423. doi:10.1089/088282403322396208. 14583155

[49] Tumpey TM, Basler CF, Aguilar PV, Zeng H, Solórzano A, Swayne DE, Cox NJ, Katz JM, Taubenberger JK, Palese P, García-Sastre A. 2005. Characterization of the reconstructed 1918 Spanish influenza pandemic virus. Science 310:77–80. doi:10.1126/science.1119392. 16210530

[50] Chaipan C, Kobasa D, Bertram S, Glowacka I, Steffen I, Tsegaye TS, Takeda M, Bugge TH, Kim S, Park Y, Marzi A, Pöhlmann S. 2009. Proteolytic activation of the 1918 influenza virus hemagglutinin. J Virol 83:3200–3211. doi:10.1128/JVI.02205-08. 19158246 PMC2655587

[51] Schrauwen EJA, Bestebroer TM, Munster VJ, de Wit E, Herfst S, Rimmelzwaan GF, Osterhaus ADME, Fouchier RAM. 2011. Insertion of a multibasic cleavage site in the haemagglutinin of human influenza H3N2 virus does not increase pathogenicity in ferrets. J Gen Virol 92:1410–1415. doi:10.1099/vir.0.030379-0. 21346026 PMC3168280

[52] Froggatt HM, Heaton NS. 2022. Nonrespiratory sites of influenza-associated disease: mechanisms and experimental systems for continued study. FEBS J 289:4038–4060. doi:10.1111/febs.16363. 35060315 PMC9300775

[53] Bryche B, Frétaud M, Saint-Albin Deliot A, Galloux M, Sedano L, Langevin C, Descamps D, Rameix-Welti M-A, Eléouët J-F, Le Goffic R, Meunier N. 2020. Respiratory syncytial virus tropism for olfactory sensory neurons in mice. J Neurochem 155:137–153. doi:10.1111/jnc.14936. 31811775

[54] Bryche B, St Albin A, Murri S, Lacôte S, Pulido C, Ar Gouilh M. 2020. Massive transient damage of the olfactory epithelium associated with infection of sustentacular cells by SARS-CoV-2 in golden Syrian hamsters. Brain Behav Immun 89:579–586. doi:10.1016/j.bbi.2020.06.032. 32629042 PMC7332942

[55] Li S, Bai S, Qin X, Zhang J, Irwin DM, Zhang S, Wang Z. 2019. Comparison of whole embryonic development in the duck (Anas platyrhynchos) and goose (Anser cygnoides) with the chicken (Gallus gallus). Poult Sci 98:3278–3291. doi:10.3382/ps/pez133. 30941418

[56] Figueroa T, Bessière P, Coggon A, Bouwman KM, van der Woude R, Delverdier M, Verheije MH, de Vries RP, Volmer R. 2020. The microbiota contributes to the control of highly pathogenic H5N9 influenza virus replication in ducks. J Virol 94:e00289-20. doi:10.1128/JVI.00289-20. 32102887 PMC7199410

[57] Smith J, Sadeyen J-R, Butter C, Kaiser P, Burt DW. 2015. Analysis of the early immune response to infection by infectious bursal disease virus in chickens differing in their resistance to the disease. J Virol 89:2469–2482. doi:10.1128/JVI.02828-14. 25505077 PMC4325706

[58] Arathy DS, Nair S, Soman SS, Issac A, Sreekumar E. 2011. Functional characterization of the CC chemokine RANTES from Pekin duck (Anas platyrhynchos). Dev Comp Immunol 35:142–150. doi:10.1016/j.dci.2010.09.005. 20850473

